# From stress to resource: embodied cognitive pathways linking dance competence and psychological resilience

**DOI:** 10.3389/fpsyg.2026.1906128

**Published:** 2026-08-19

**Authors:** Ziyu Zheng, Ekasak Hengsuko, Wiradee Eakronnarongchai, Onsuda Wiangthongsarat

**Affiliations:** 1 Udon Thani Rajabhat University, Udon Thani, Thailand; 2 School of Music and Dance, Heze University, Heze, Shandong, China

**Keywords:** bodily self-representation, cognitive flexibility, dance education, embodied cognition, perceived dance competence, psychological resilience

## Abstract

**Background:**

Dance training presents a distinctive stress–resource context in which physical, cognitive, and emotional demands co-exist with potential adaptive benefits. However, the body-mediated cognitive processes through which perceived dance competence relates to psychological resilience remain underexplored. This study examined whether perceived dance competence was associated with psychological resilience through dimensions of embodied cognition among undergraduate dance students.

**Methods:**

A total of 303 Chinese undergraduate dance students completed self-report measures of perceived dance competence, embodied cognition (motor control, negative experience processing, bodily self-representation, cognitive flexibility, and mind–body integration), and psychological resilience. Confirmatory factor analyses and structural equation modeling with bias-corrected bootstrap estimation were used to examine the proposed association model.

**Results:**

Perceived dance competence was positively associated with all five embodied cognition dimensions. Cognitive flexibility (β = 0.356, p < 0.001) and bodily self-representation (β = 0.301, p < 0.001) showed the strongest positive associations with psychological resilience, whereas negative experience processing and mind–body integration were not significant predictors. The total indirect association between perceived dance competence and psychological resilience through embodied cognition dimensions was significant (β = 0.287, 95% CI [0.20, 0.39], p < 0.001), while the direct association was weak and non-significant (β = –0.012, p = 0.794).

**Conclusion:**

These findings suggest that body-mediated cognitive processes, particularly cognitive flexibility and bodily self-representation, may be relevant to psychological resilience in dance education contexts. However, the cross-sectional design, self-report methodology, newly developed or adapted instruments, and suboptimal incremental model fit warrant cautious interpretation. Future longitudinal, experimental, and measurement-validation studies are needed to test the temporal and causal assumptions of the proposed model.

## 1 Introduction

### 1.1 The double-edged sword of dance training

Dance training provides a distinctive context for examining psychological adaptation in movement-based education. Unlike many forms of physical activity, dance requires the continuous coordination of technical control, esthetic expression, bodily awareness, emotional communication, rhythmic sensitivity, and performance under evaluation. These characteristics make dance a highly embodied learning environment in which physical, cognitive, affective, and social processes are closely intertwined.

At the same time, dance training should not be understood as uniformly beneficial. Undergraduate dance students are often exposed to demanding technical standards, repeated correction, body-related evaluation, intensive rehearsal schedules, injury concerns, and performance pressure. These experiences may be associated with psychological strain, including anxiety, fatigue, emotional exhaustion, and body image concerns (Walker and Nordin-Bates, 2010; Rice et al., 2016; Robazza et al., 2024; Dwarika and Haraldsen, 2023). Thus, dance training can be understood as a stress–resource context: it may be associated with both psychological demands and adaptive psychological resources.

This dual character is particularly important for understanding psychological resilience among dance students. On the one hand, intensive training, evaluative pressure, and bodily self-scrutiny may be associated with reduced psychological resources. On the other hand, sustained participation in dance practice may also be related to self-regulation, emotional expression, perceived competence, and adaptive coping capacities (Fontanesi and DeSouza, 2021; Christensen et al., 2017; Klaperski-van der Wal et al., 2025). However, the processes through which dance-related self-perceptions are associated with psychological resilience remain insufficiently specified.

Importantly, the present study does not treat objective dance training exposure as the focal predictor. Objective training exposure refers to indicators such as years of training, weekly practice hours, or performance history. In contrast, the present study focuses on perceived dance competence, defined as dancers’ subjective evaluation of their technical, expressive, musical, learning-related, and adaptive capabilities within dance practice. This distinction is necessary because training exposure and perceived competence are conceptually different. Two students with similar training histories may evaluate their dance-related capability differently, and such self-perceptions may be more closely associated with psychological adaptation than training duration alone. Therefore, this study examines whether perceived dance competence is associated with psychological resilience through specific dimensions of embodied cognition.

### 1.2 Psychological resilience in the performing arts

Psychological resilience is commonly defined as the capacity to maintain, recover, or regain adaptive functioning when facing stress, challenge, or adversity (Fletcher and Sarkar, 2012). In sport psychology, resilience has often been examined in relation to competitive pressure, coping with setbacks, performance stability, and recovery from failure (Sarkar and Fletcher, 2014). These perspectives provide a useful foundation for understanding adaptation in high-demand performance contexts.

However, the resilience demands faced by dancers differ in important ways from those typically emphasized in competitive sport. Competitive sport is often organized around external comparison, winning and losing, and measurable performance outcomes. Dance, by contrast, places strong emphasis on esthetic expression, bodily self-construction, emotional communication, and the continuous refinement of movement quality (Quested and Duda, 2011; Walker and Nordin-Bates, 2010; Robazza et al., 2024). Although dance students also experience auditions, examinations, competitions, and teacher evaluation, the psychological demands of dance are often strongly tied to the body as both an expressive medium and an object of evaluation.

From this perspective, resilience in dance contexts may involve more than general coping ability. It may also depend on how dancers perceive, regulate, and represent their bodies under demanding conditions. A dancer’s ability to adjust movement after feedback, reinterpret bodily discomfort, maintain confidence in the body under evaluation, and adapt to changing rehearsal or performance situations may be closely associated with psychological adaptation. Stress-adaptation perspectives suggest that repeated exposure to manageable stressors may be related to adaptive functioning when individuals develop effective regulatory strategies (Meichenbaum, 2007). Dance training may provide such experiences through technical practice, rehearsal feedback, improvisation, and performance, although the present cross-sectional study cannot directly test developmental or causal processes.

Not all dancers respond to training demands in the same way. Similar levels of training exposure may be associated with fatigue or distress in some individuals but with confidence, adaptive coping, or psychological growth in others (Rice et al., 2016; Robazza et al., 2024). This variability suggests that the relationship between dance-related competence and resilience cannot be adequately explained by a simple “more training equals more benefit” assumption. Instead, it is necessary to examine the psychological and body-mediated processes through which perceived dance competence is associated with resilience.

### 1.3 Embodied cognition as a conceptual lens

Embodied cognition provides a useful conceptual framework for examining how bodily experience may be associated with cognitive, affective, and psychological functioning. However, embodied cognition should not be treated as a single unified theory. Rather, it refers to a family of theoretical approaches that emphasize the role of the body in perception, action, emotion, cognition, and self-understanding (Wilson, 2002; Shapiro, 2019). These approaches include grounded cognition, conceptual metaphor theory, sensorimotor accounts, and enactive perspectives.

The present study adopts the enactive perspective as a conceptual lens rather than as a directly tested theoretical model. From an enactive viewpoint, cognition emerges through ongoing interactions among the body, action, and environment (Varela et al., 1991). The body is not merely an instrument for executing movement; it participates in how individuals perceive situations, regulate responses, and construct meaning through action. This perspective is particularly relevant to dance because dance practice requires continuous coordination among perception, movement execution, emotion, rhythm, space, and environmental feedback.

In dance learning, perceived competence is closely tied to bodily action. Dancers learn not only to perform movements but also to sense movement quality, anticipate bodily adjustments, respond to spatial and musical cues, and interpret feedback from teachers, peers, and audiences. These processes are consistent with the idea that cognition is shaped through perception–action cycles (Varela et al., 1991; Barsalou, 2008; Shapiro, 2019). Thus, an embodied cognition framework may help explain why perceived dance competence is not merely a technical self-evaluation but may also be associated with broader psychological functioning.

The connection between embodied cognition and psychological resilience can be further clarified through resilience theory. Contemporary resilience perspectives emphasize dynamic adaptation rather than fixed personality traits. Resilience is understood as emerging from the interaction of multiple protective factors, including cognitive flexibility, self-regulation, emotional regulation, perceived competence, and contextual support (Fletcher and Sarkar, 2012; Meichenbaum, 2007). From this standpoint, body-mediated cognitive processes may represent one set of resources associated with adaptation under stress.

Accordingly, embodied cognition may be relevant to resilience because it links bodily experience with cognitive appraisal, emotional regulation, and self-representation. For example, the perceived ability to regulate movement may be associated with a sense of control; coherent bodily self-representation may be related to confidence under evaluation; and cognitive flexibility may help individuals reinterpret mistakes, discomfort, or performance setbacks (Niedenthal, 2007; Koch et al., 2019; Price and Hooven, 2018). These statements are theoretical propositions rather than causal claims. The present cross-sectional study examines whether the observed pattern of associations is consistent with this proposed body-mediated account.

### 1.4 Multidimensional embodied cognition in dance

Embodied cognition in dance is unlikely to operate as a single homogeneous process. Dance practice involves multiple body-based cognitive functions, ranging from movement regulation to self-representation and flexible adaptation. Previous research on body awareness, interoception, self-representation, and mind–body processes suggests that bodily experience includes multiple distinguishable but related dimensions (Mehling et al., 2009; Mehling et al., 2012; Price and Hooven, 2018). Therefore, the present study conceptualizes embodied cognition as a multidimensional construct.

First, motor control and body awareness refer to the perceived ability to regulate bodily movement, coordinate posture, and adjust movement execution in real time. In dance, this dimension is closely related to technical control, balance, spatial orientation, movement precision, and responsiveness to bodily feedback. Long-term dance practice may be associated with heightened sensitivity to posture, movement trajectory, spatial positioning, and bodily boundaries (Bläsing et al., 2012; Christensen et al., 2017; Canaipa et al., 2021).

Second, negative experience processing refers to the way individuals perceive and process bodily discomfort, tension, fatigue, or negative bodily experiences. This dimension is important because bodily awareness is not always adaptive. Heightened attention to bodily signals may be associated with regulation in some contexts but with worry, discomfort, or distress in others. In the present study, this dimension is treated as a distinct facet of embodied cognition rather than as a simple indicator of positive functioning.

Third, bodily self-representation refers to the cognitive and affective representation of one’s body as coherent, capable, and integrated into the self. In dance, the body is both the medium of performance and an object of evaluation. Therefore, how dancers represent their bodies may be associated with confidence, self-continuity, and psychological adaptation under evaluative conditions (Quested and Duda, 2011; Walker and Nordin-Bates, 2010).

Fourth, cognitive flexibility refers to the capacity to adjust cognitive strategies, reinterpret situations, and adapt responses when facing changing demands. In dance practice, this may involve modifying movement after feedback, adapting to unfamiliar choreography, responding to performance errors, or transforming frustration into further learning. Movement-based practice and dance experience have been discussed in relation to adaptive cognition, creativity, and flexible response patterns (Coubard et al., 2011; Cross and Ticini, 2012; Batson and Sentler, 2017; Koch et al., 2019). However, direct evidence for cognitive flexibility in dance populations remains limited and requires further empirical clarification.

Fifth, mind–body integration refers to the perceived coherence between bodily states and mental awareness. In dance, individuals often experience movement, attention, and emotion as integrated rather than separate. However, the relationship between mind–body integration and psychological resilience may not be straightforward. Strong perceived integration may reflect adaptive coherence in some situations, but its psychological significance may depend on training stage, emotional context, individual differences, or performance demands (Mehling et al., 2012; Price and Hooven, 2018).

These dimensions are theoretically related but conceptually distinct. Body awareness concerns the perception and regulation of bodily signals; interoception concerns sensitivity to internal physiological states; bodily self-representation concerns how the body is cognitively and affectively represented as part of the self; cognitive flexibility concerns adaptive reinterpretation and response adjustment; and mind–body integration concerns perceived coherence between bodily and mental states. Clarifying these distinctions is necessary because the present study examines whether different embodied cognition dimensions show different patterns of association with psychological resilience.

### 1.5 Research gaps and rationale

Although previous research has examined the psychological benefits and risks of physical activity, sport participation, and dance, several gaps remain.

First, many studies have examined associations between physical activity or training and psychological outcomes, but fewer have specified how body-mediated cognitive processes may be associated with resilience in performing arts contexts. A substantial literature in psychology, neuroscience, motor cognition, and sport psychology has examined mechanisms linking movement and psychological functioning (Pesce et al., 2015; Tomporowski and Pesce, 2019). Nevertheless, the specific pathways through which dance—as an esthetic, expressive, and body-centered practice—is associated with psychological resilience remain underexplored.

Second, existing work often focuses on general psychological variables such as self-efficacy, motivation, anxiety, or well-being. These constructs are important, but they may not fully capture the body-mediated processes through which dancers adapt to training and performance demands. A multidimensional embodied cognition framework may help identify more specific correlates of resilience, such as motor control, bodily self-representation, and cognitive flexibility (Mehling et al., 2012; Shapiro, 2019; Koch et al., 2019).

Third, previous research sometimes treats dance or physical activity as uniformly beneficial, which may overlook the dual nature of dance training. Dance may be associated with positive psychological resources, but it may also involve body-related pressure, emotional exhaustion, injury concerns, and evaluative stress. Therefore, research should avoid assuming that greater training exposure automatically produces better psychological outcomes. Instead, it is necessary to examine how dancers’ subjective competence evaluations and body-mediated cognitive processes are associated with resilience.

Fourth, the relationship between perceived dance competence and psychological resilience may not be direct. Perceived dance competence may be associated with resilience primarily through specific embodied cognition dimensions rather than through technical self-evaluation alone. However, this possibility has received limited empirical attention, particularly among undergraduate dance students.

### 1.6 The present study

The present study investigates the associations among perceived dance competence, multidimensional embodied cognition, and psychological resilience in a sample of Chinese undergraduate dance students. Perceived dance competence is examined as the focal predictor because it captures dancers’ subjective evaluation of their dance-related capabilities rather than objective training exposure. Embodied cognition is examined as a multidimensional set of body-mediated cognitive, affective, and self-representational processes that may statistically account for the association between perceived dance competence and psychological resilience.

**Figure 1:**
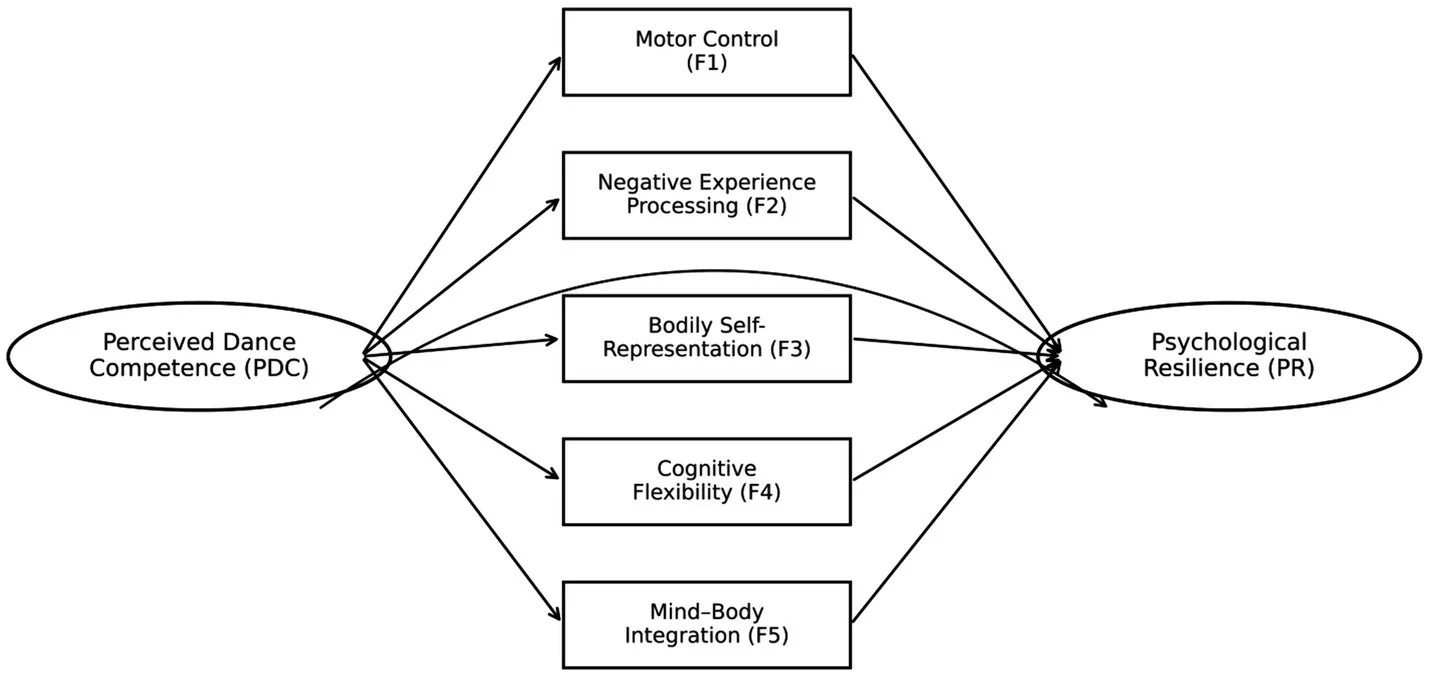
Conceptual model of the proposed cross-sectional indirect associations among perceived dance competence, embodied cognition dimensions, and psychological resilience.

Given the cross-sectional design, the study does not test causal hypotheses or establish temporal precedence. Instead, it evaluates whether the observed pattern of associations is consistent with the theoretical proposition that specific embodied cognition dimensions are associated with the link between perceived dance competence and psychological resilience. The proposed conceptual model is presented in Figure 1.

The study addresses the following research questions:

RQ1. Is perceived dance competence associated with multiple dimensions of embodied cognition?

RQ2. Do different dimensions of embodied cognition show differential associations with psychological resilience?

RQ3. Is the association between perceived dance competence and psychological resilience statistically accounted for by dimensions of embodied cognition?

RQ4. Does perceived dance competence show a direct association with psychological resilience after accounting for embodied cognition dimensions?

By addressing these questions, the present study aims to provide preliminary cross-sectional evidence on the role of embodied cognition in the relationship between perceived dance competence and psychological resilience. The findings are intended to inform future longitudinal, experimental, and measurement-validation studies that can more directly test the temporal and causal assumptions of the proposed theoretical model.

## 2 Methods

### 2.1 Participants and procedure

An *a priori* power analysis was conducted using G*Power 3.1 (Faul et al., 2009) to estimate the minimum sample size required for the planned analyses. Assuming a medium effect size, f^2^ = 0.15, a significance level of *α* = 0.05, and statistical power of 1 − *β* = 0.95, the minimum required sample size was 210 participants. Considering the additional sample size requirements associated with latent variable modeling and bootstrap estimation, 303 undergraduate dance students were included in the final analytic sample.

Participants were recruited from the School of Music and Dance at Heze University, Shandong Province, China. All participants were undergraduate students majoring in dance and had received systematic dance training for more than 3 years. Their dance backgrounds included Chinese classical dance, Chinese folk dance, ballet, and contemporary dance. The average accumulated training experience was 8.47 years (SD = 2.63), and the average weekly training duration was 17.8 h (SD = 5.42). Training activities included technique classes, repertoire rehearsals, improvisational practice, physical conditioning, and stage performance preparation.

Data collection was conducted during a regular academic training period rather than immediately before major performances, final examinations, or graduation assessments, in order to reduce potential fluctuations associated with acute evaluative stress. Questionnaires were administered in a centralized in-person setting. Participants completed the survey collectively under standardized instructions provided by the research team. Participation was voluntary, and no course credit, teacher evaluation, or performance assessment was linked to participation.

The research protocol was approved by the Research Ethics Committee of Udon Thani Rajabhat University, Thailand, approval no. 0622.7/629. Permission to recruit participants and administer the survey was also obtained from the School of Music and Dance at Heze University, where data collection took place. All procedures were conducted in accordance with the Declaration of Helsinki (World Medical Association, 2024). Before participation, all students were informed of the research purpose, voluntary participation policy, confidentiality procedures, and their right to withdraw at any time without penalty. Written informed consent was obtained from all participants. For participants under the age of 18, additional written informed consent was obtained from their legal guardians. All responses were anonymized using coded identifiers and stored in encrypted files accessible only to the research team.

### 2.2 Translation and cross-cultural adaptation

Because the study was conducted with Chinese undergraduate dance students, all instruments were translated and culturally adapted before formal data collection. The translation and adaptation procedure followed standard back-translation principles (Brislin, 1970).

First, the original English items were translated into simplified Chinese by a bilingual researcher with training in psychology and dance education. Second, an independent bilingual expert who was blind to the original English version back-translated the Chinese version into English. Third, the research team compared the back-translated version with the original English version to identify discrepancies in meaning, wording, and construct representation. Fourth, a review panel consisting of three dance education experts and one psychology professor evaluated the Chinese version for content relevance, cultural appropriateness, readability, and suitability for undergraduate dance students.

Finally, a pilot study was conducted with 136 undergraduate dance students. During the pilot stage, cognitive interviews were conducted with 15 participants to examine whether the items were understandable, culturally appropriate, and consistent with the intended constructs. Minor wording adjustments were made based on participant feedback. No substantial linguistic or cultural adaptation problems were identified. The final Chinese version was used for the formal survey.

This procedure was particularly important because the ethical approval was granted by a Thai university, whereas data were collected from a Chinese undergraduate sample. Therefore, the instruments were not administered in English or Thai during formal data collection; they were administered in simplified Chinese after translation, back-translation, expert review, and pilot testing.

### 2.3 Measures

#### 2.3.1 Perceived dance competence

Perceived dance competence was assessed using the Dance Competence Assessment and Practice Scale (D-CAPS). In the present study, perceived dance competence was defined as dancers’ subjective evaluation of their dance-related technical, expressive, learning-related, musical, and adaptive capabilities. This construct is conceptually distinct from objective training exposure, such as years of training, weekly practice hours, or performance history.

The D-CAPS contains 20 items across five dimensions: technical execution and body control, stage performance and emotional expression, movement learning and bodily imitation, musical structure understanding and choreographic creativity, and situational adaptability and improvisational regulation. Each dimension contains four items. Items are rated on a five-point Likert scale ranging from 1 = strongly disagree to 5 = strongly agree, with higher scores indicating higher perceived dance competence.

Representative items include: “I can execute technically demanding movements required in my dance style with precision and clarity,” “I can accurately convey the emotional content of a piece through bodily expression,” and “I can quickly adjust my movement amplitude when performing under different floor or lighting conditions.”

In the pilot sample (*N* = 136), exploratory factor analysis supported a five-factor structure, KMO = 0.926, Bartlett’s *χ*^2^ = 2398.030, df = 300, *p* < 0.001, explaining 68.763% of the total variance. In the formal sample (*N* = 303), internal consistency coefficients were satisfactory across the five dimensions: technical execution and body control *α* = 0.855, stage performance and emotional expression *α* = 0.931, movement learning and bodily imitation *α* = 0.848, musical structure understanding and choreography *α* = 0.889, and situational adaptability and improvisational regulation *α* = 0.891.

Because the D-CAPS was developed for the present research program and has not yet been independently validated in published studies, the findings based on this measure should be interpreted as preliminary. Full confirmatory factor analysis results, including standardized factor loadings, standard errors, composite reliability (CR), and average variance extracted (AVE), are reported in Supplementary Table S1.

#### 2.3.2 Embodied cognition

Embodied cognition was assessed using the Embodied Cognition Assessment Scale (ECAS), which was developed to measure body-mediated cognitive, affective, and self-representational processes in dance contexts. The initial item pool contained 40 items. After expert review, item analysis, and exploratory factor analysis in the pilot sample, the final version retained 27 items.

The ECAS includes five dimensions: motor control and action efficacy, negative experience processing, bodily self-representation, cognitive flexibility, and mind–body integration. Motor control and action efficacy refers to perceived capacity for movement regulation and bodily control. Negative experience processing refers to the interpretation and regulation of bodily discomfort, tension, or negative bodily experience. Bodily self-representation refers to the cognitive and affective representation of the body as coherent and capable. Cognitive flexibility refers to the ability to adjust cognition and responses under changing demands. Mind–body integration refers to perceived coherence between bodily states and mental awareness.

All items are rated on a five-point Likert scale ranging from 1 = strongly disagree to 5 = strongly agree. Reverse-worded items were recoded before analysis so that higher scores consistently indicated higher levels of the intended dimension.

Representative items include: “Controlling bodily movements is easy for me” for motor control and action efficacy, “I am good at analyzing and evaluating different situations and contexts” for cognitive flexibility, and “My awareness and my body feel integrated as one” for mind–body integration.

In the pilot sample (*N* = 136), the data were suitable for factor analysis, KMO = 0.827, Bartlett’s *χ*^2^(780) = 3468.905, *p* < 0.001. The five-factor structure explained 58.42% of the total variance. In the formal sample (*N* = 303), internal consistency coefficients were as follows: motor control and action efficacy *α* = 0.894, negative experience processing *α* = 0.759, bodily self-representation *α* = 0.900, cognitive flexibility *α* = 0.797, and mind–body integration *α* = 0.638.

The relatively lower reliability coefficient for the mind–body integration dimension should be noted. This dimension contains only three items and captures a relatively implicit experiential construct; therefore, results involving this dimension should be interpreted cautiously. Because the ECAS is a newly developed measure, the present study provides only initial psychometric evidence. Independent validation in future samples is required. Full CFA results, including standardized loadings, standard errors, CR, and AVE, are reported in Supplementary Table S2.

#### 2.3.3 Psychological resilience

Psychological resilience was assessed using the University Student Training Situational Resilience Scale (USTRS). The USTRS was developed for the present research program by adapting the theoretical structure of psychological resilience to the training and performance context of undergraduate dance students. It was designed to assess psychological adaptation and regulatory capacity in dance-related training situations.

The scale contains 26 items across five dimensions: autonomous agency, situational pressure, competence satisfaction, emotion regulation, and relational support. Items are rated on a five-point Likert scale ranging from 1 = strongly disagree to 5 = strongly agree. Reverse-coded items were recoded before analysis so that higher scores indicated higher levels of psychological resilience or adaptive functioning.

Representative items include: “I can adjust my emotions within a short period of time,” “I feel competent in my major or training activities,” and “I can obtain support from others when I encounter difficulties in training.” Items reflecting pressure or negative experience were reverse-coded when required.

In the pilot sample (*N* = 136), Bartlett’s test of sphericity was significant, *χ*^2^ = 2178.937, df = 276, *p* < 0.001, and exploratory factor analysis supported a five-factor structure consistent with the theoretical framework. In the formal sample (*N* = 303), internal consistency coefficients were acceptable across dimensions: autonomous agency *α* = 0.857, situational pressure *α* = 0.885, competence satisfaction *α* = 0.865, emotion regulation *α* = 0.848, and relational support *α* = 0.784.

Because the USTRS was adapted for the specific context of undergraduate dance training and has not yet been independently validated against established resilience instruments, findings involving this measure should be considered preliminary (Connor and Davidson, 2003). The present study did not include an external criterion measure of resilience; therefore, criterion validity could not be examined. Full CFA results, including standardized loadings, standard errors, CR, and AVE, are reported in Supplementary Table S3.

#### 2.3.4 Covariates

Several demographic and training-related variables were included as covariates because they may be associated with perceived dance competence or psychological resilience. These variables included gender, cumulative years of training, weekly training frequency, performance experience, and number of dance styles learned.

Training duration and training frequency were treated as indicators of objective training exposure, whereas perceived dance competence was treated as a subjective self-evaluation of dance-related capability. This distinction was central to the present study because the theoretical model focused on perceived competence rather than training volume.

### 2.4 Data analysis

All analyses were conducted using SPSS Statistics 27 and AMOS 26.0. Because the data were cross-sectional and self-reported, all structural analyses were interpreted as tests of theoretically specified association patterns rather than tests of causal mechanisms or temporal ordering.

First, data screening was conducted to examine missing values, normality, and multivariate outliers. The proportion of missing responses for individual items was below 2%. Little’s MCAR test was used to examine the pattern of missing data. Because the proportion of missing data was very low and missingness was random, listwise deletion was applied. Univariate skewness and kurtosis were examined to assess distributional assumptions. Multivariate outliers were evaluated using Mahalanobis distance based on the seven composite study variables: perceived dance competence, five embodied cognition dimensions, and psychological resilience. The critical value was set at *p* < 0.001, *χ*^2^(7) = 24.32.

Second, descriptive statistics and Pearson correlations were calculated for perceived dance competence, the five embodied cognition dimensions, and psychological resilience. These analyses provided preliminary information about the direction and magnitude of bivariate associations.

Third, confirmatory factor analysis (CFA) was conducted to evaluate the measurement structure of the three instruments. The CFA analyses examined the five-factor structure of D-CAPS, the five-factor structure of ECAS, and the five-factor structure of USTRS. Model fit was evaluated using multiple indices, including *χ*^2^/df, CFI, TLI, RMSEA, and SRMR. Following conventional guidelines, CFI and TLI values around 0.90 or above were treated as indicating acceptable fit, RMSEA values below 0.08 as indicating reasonable fit, and SRMR values below 0.08 as indicating acceptable residual fit. When fit indices did not reach conventional standards, the model was described as marginal or suboptimal rather than fully acceptable.

Fourth, convergent validity was evaluated using standardized factor loadings, composite reliability (CR), and average variance extracted (AVE). Discriminant validity was examined using the heterotrait–monotrait ratio (HTMT). Because the instruments were newly developed or adapted, these analyses were treated as initial psychometric evidence rather than definitive validation.

Fifth, the proposed association model was tested using structural equation modeling. Perceived dance competence was specified as the focal predictor, the five embodied cognition dimensions were specified as parallel statistical mediators, and psychological resilience was specified as the outcome variable. The model estimated the direct association between perceived dance competence and psychological resilience, as well as the indirect associations through each embodied cognition dimension.

Indirect associations were tested using a bias-corrected bootstrap procedure with 5,000 resamples. Ninety-five percent confidence intervals were reported. An indirect association was considered statistically significant when the confidence interval did not include zero. Because the study design was cross-sectional, these estimates are referred to as indirect associations rather than evidence of causal mediation.

Sixth, robustness checks were conducted by re-estimating the model after including covariates: gender, cumulative years of training, weekly training frequency, performance experience, and number of dance styles learned. The purpose of these analyses was to examine whether the core association pattern remained stable after accounting for demographic and training-related background variables.

Finally, given concerns regarding model complexity and measure overlap, no moderation hypothesis was retained in the main model. The previous motor-control moderation analysis was treated as exploratory and is reported only in the Supplementary material. This decision was made because motor control is one dimension of embodied cognition and therefore conceptually overlaps with the proposed mediating structure. Retaining it as a moderator in the main model would risk introducing theoretical ambiguity.

## 3 Results

### 3.1 Data screening and preliminary analyses

The final analytic sample consisted of 303 undergraduate dance students. The proportion of missing responses for individual items was below 2%. Little’s MCAR test indicated that the missing data were completely at random, *χ*^2^(2138) = 2187.45, *p* = 0.24. Given the low proportion of missing data and the random missingness pattern, listwise deletion was applied.

Univariate skewness values ranged from −0.98 to 0.56, and kurtosis values ranged from −0.59 to 1.88. These values were within commonly accepted thresholds for maximum likelihood estimation, indicating no serious deviation from univariate normality. Multivariate outliers were examined using Mahalanobis distance based on the seven composite study variables: perceived dance competence, five embodied cognition dimensions, and psychological resilience. The critical value was set at *p* < 0.001, *χ*^2^(7) = 24.32. No case exceeded this threshold, suggesting that no extreme multivariate outliers were present.

To assess the potential influence of common method variance, Harman’s single-factor test was conducted (Podsakoff et al., 2003). The unrotated exploratory factor analysis indicated that the first factor accounted for 28.4% of the total variance, below the commonly used 40% threshold. In addition, a single-factor confirmatory model demonstrated poor fit, *χ*^2^/df = 8.72, CFI = 0.42. These results suggest that common method variance was unlikely to fully account for the observed associations. However, Harman’s test is limited and should not be treated as definitive evidence against common method bias. Given the cross-sectional and single-source design, common method variance may still have inflated the magnitude of some associations.

Descriptive statistics and correlations are presented in Table 1. Perceived dance competence was positively correlated with motor control, *r* = 0.65, *p* < 0.01, negative experience processing, *r* = 0.20, *p* < 0.01, bodily self-representation, *r* = 0.27, *p* < 0.01, cognitive flexibility, *r* = 0.33, *p* < 0.01, mind–body integration, *r* = 0.65, *p* < 0.01, and psychological resilience, *r* = 0.18, *p* < 0.01. Psychological resilience was positively correlated with motor control, *r* = 0.51, *p* < 0.01, negative experience processing, *r* = 0.18, *p* < 0.01, bodily self-representation, *r* = 0.54, *p* < 0.01, cognitive flexibility, *r =* 0.58, *p* < 0.01, and mind–body integration, *r* = 0.34, *p* < 0.01. These correlations provided preliminary support for examining indirect association patterns among the core variables.

**Table 1:** Descriptive statistics and correlation matrix of study variables (*N* = 303).

Variable	*M*	SD	1	2	3	4	5	6	7
1. Perceived dance competence	3.12	0.58	–						
2. Motor control F1	3.28	0.65	0.65**	–					
3. Negative experience processing F2	2.89	0.71	0.20**	0.24**	–				
4. Bodily self-representation F3	3.45	0.52	0.27**	0.43**	0.34**	–			
5. Cognitive flexibility F4	3.62	0.48	0.33**	0.56**	0.11	0.48**	–		
6. Mind–body integration F5	3.25	0.54	0.65**	0.37**	0.53**	0.31**	0.39**	–	
7. Psychological resilience	3.51	0.61	0.18**	0.51**	0.18**	0.54**	0.58**	0.34**	–

**p < 0.01.

### 3.2 Confirmatory factor analysis and initial psychometric evidence

Before testing the structural association model, confirmatory factor analyses were conducted to evaluate the measurement properties of the three instruments used in the study: the Dance Competence Assessment and Practice Scale (D-CAPS), the Embodied Cognition Assessment Scale (ECAS), and the University Student Training Situational Resilience Scale (USTRS). Because these instruments were newly developed or adapted for the present research program, the CFA results should be interpreted as initial psychometric evidence rather than as independent validation.

For the ECAS, the initial five-factor model showed marginal fit: *χ*^2^(314) = 883.762, *χ*^2^/df = 2.815, CFI = 0.859, TLI = 0.842, RMSEA = 0.078, 90% CI [0.071, 0.085], SRMR = 0.060. After allowing one theoretically justified residual covariance between conceptually overlapping items with shared action-regulation content, model fit improved: *χ*^2^(313) = 798.221, *χ*^2^/df = 2.550, CFI = 0.882, TLI = 0.867, RMSEA = 0.072, 90% CI [0.065, 0.079]. Although RMSEA and SRMR indicated reasonable fit, CFI and TLI remained below the conventional 0.90 threshold (Hu and Bentler, 1999). Therefore, the ECAS measurement model was treated as providing preliminary but not fully satisfactory evidence of structural validity.

All standardized factor loadings for the ECAS were statistically significant, *p* < 0.001, and ranged from 0.56 to 0.88. Composite reliability values ranged from 0.76 to 0.90, and average variance extracted (AVE; Fornell and Larcker, 1981) values ranged from 0.42 to 0.65. The negative experience processing dimension showed AVE below 0.50, indicating that convergent validity for this dimension was weaker than ideal. The mind–body integration dimension contained only three items and showed relatively low internal consistency; therefore, findings involving this dimension should be interpreted cautiously.

For the D-CAPS, the five-factor CFA model demonstrated good fit: *χ*^2^(160) = 309.929, *χ*^2^/df = 1.937, CFI = 0.969, TLI = 0.963, RMSEA = 0.056, 90% CI [0.048, 0.064], SRMR = 0.040. Standardized factor loadings ranged from 0.68 to 0.87, CR values ranged from 0.83 to 0.88, and AVE values ranged from 0.54 to 0.65. These results provided initial support for the five-dimensional structure of perceived dance competence in the present sample.

For the USTRS, the five-factor CFA model showed mixed fit: *χ*^2^(289) = 850.068, *χ*^2^/df = 2.941, CFI = 0.871, TLI = 0.855, RMSEA = 0.080, 90% CI [0.073, 0.087], SRMR = 0.070. RMSEA and SRMR were within or near the range of reasonable fit, but CFI and TLI were below the conventional 0.90 threshold. Standardized factor loadings ranged from 0.60 to 0.85, CR values ranged from 0.80 to 0.85, and AVE values ranged from 0.45 to 0.59. The AVE values for autonomous agency, situational pressure, and relational support were slightly below 0.50, suggesting that convergent validity for these dimensions requires further examination.

Discriminant validity among the five embodied cognition dimensions was assessed using the heterotrait–monotrait ratio (HTMT; Henseler et al., 2015). All HTMT values were below the conservative threshold of 0.85, ranging from 0.31 to 0.72, indicating that the five dimensions captured related but distinguishable facets of embodied cognition. Nevertheless, because all measures were self-reported and two of the three scales were newly developed while one was contextually adapted, the psychometric evidence should be regarded as preliminary. Full item-level CFA results for D-CAPS, ECAS, and USTRS, including standardized loadings, standard errors, CR, and AVE, are reported in Supplementary Tables S1–S3 and Table 2.

**Table 2:** Summary of confirmatory factor analysis results.

Measure	*χ*^2^\chi^^2^*χ*^2^	*df*	*χ*^2^/df\chi^^2^/df *χ*^2^/df	CFI	TLI	RMSEA	SRMR
ECAS initial five-factor model	883.762	314	2.815	0.859	0.842	0.078	0.06
ECAS modified five-factor model	798.221	313	2.55	0.882	0.867	0.072	0.06
D-CAPS five-factor model	309.929	160	1.937	0.969	0.963	0.056	0.04
USTRS five-factor model	850.068	289	2.941	0.871	0.855	0.08	0.07

ECAS, Embodied Cognition Assessment Scale; D-CAPS, Dance Competence Assessment and Practice Scale; USTRS, University Student Training Situational Resilience Scale.

### 3.3 Structural model fit and model specification

The hypothesized structural model specified perceived dance competence as the focal predictor, the five embodied cognition dimensions as parallel indirect association components, and psychological resilience as the outcome variable. Given the theoretical aim of distinguishing the specific roles of different embodied cognition dimensions, a first-order parallel model was used as the primary model.

The first-order parallel model showed acceptable absolute fit but suboptimal incremental fit: *χ*^2^(2879) = 5645.426, *χ*^2^/df = 1.961, CFI = 0.821, TLI = 0.813, RMSEA = 0.056. Although the *χ*^2^/df and RMSEA values suggested reasonable approximation, the CFI and TLI values were below conventional standards. Therefore, the structural model should not be interpreted as providing strong confirmatory evidence for the proposed theoretical structure.

A second-order embodied cognition model was also examined to test whether the five embodied cognition dimensions could be represented by a higher-order latent construct. This model showed comparable but also suboptimal fit: χ^2^(2537) = 5346.628, *χ*^2^/df = 2.107, CFI = 0.812, TLI = 0.805, RMSEA = 0.061. The fit difference between the first-order and second-order models was small, suggesting that the two specifications captured broadly similar association structures. However, because the first-order model allowed the specific indirect associations of each embodied cognition dimension to be examined separately, it was retained as the primary analytic model.

Given the suboptimal CFI and TLI values, the following structural results are interpreted cautiously as preliminary association patterns rather than as definitive evidence of a well-fitting causal or mechanistic model (Table 3).

**Table 3:** Structural model fit comparison.

Model	*χ*^2^\chi^^2^*χ*^2^	df	*χ*^2^/df\chi^^2^/df χ^2^/df	CFI	TLI	RMSEA
M1 first-order parallel embodied cognition model	5645.426	2,879	1.961	0.821	0.813	0.056
M2 second-order embodied cognition model	5346.628	2,537	2.107	0.812	0.805	0.06

The first-order model was retained because it directly addressed the research questions concerning the differentiated roles of embodied cognition dimensions. However, both structural models showed suboptimal incremental fit; therefore, the results should be interpreted as preliminary.

### 3.4 Direct associations among perceived dance competence, embodied cognition, and psychological resilience

The first set of paths examined whether perceived dance competence was associated with the five dimensions of embodied cognition. Perceived dance competence showed significant positive associations with motor control, *β* = 0.652, *p* < 0.001, negative experience processing, *β* = 0.196, *p* < 0.001, bodily self-representation, *β* = 0.270, *p* < 0.001, cognitive flexibility, *β* = 0.330, *p* < 0.001, and mind–body integration, *β* = 0.648, *p* < 0.001. These results were consistent with RQ1, indicating that students who reported higher perceived dance competence also tended to report higher levels of multiple embodied cognition dimensions.

The second set of paths examined whether different embodied cognition dimensions were differentially associated with psychological resilience. Cognitive flexibility showed the strongest positive association with psychological resilience, *β* = 0.356, SE = 0.069, *p* < 0.001. Bodily self-representation was also positively associated with psychological resilience, *β* = 0.301, SE = 0.045, *p* < 0.001. Motor control showed a marginal positive association in the baseline model, *β* = 0.083, SE = 0.043, *p* = 0.055. In contrast, negative experience processing, *β* = 0.054, SE = 0.033, *p* = 0.102, and mind–body integration, *β* = 0.037, SE = 0.024, *p* = 0.115, were not significantly associated with psychological resilience in the baseline model.

These results were consistent with RQ2, suggesting that embodied cognition dimensions were not equally related to psychological resilience. Cognitive flexibility and bodily self-representation appeared to be the two dimensions most strongly associated with resilience in the present model. However, these findings should be interpreted as statistical associations rather than evidence that these dimensions causally enhance resilience.

The direct association between perceived dance competence and psychological resilience was weak and non-significant after the embodied cognition dimensions were included in the model, *β* = −0.012, *p* = 0.794. This result indicated that perceived dance competence was not directly associated with psychological resilience once the five embodied cognition dimensions were statistically accounted for (Table 4).

**Table 4:** Standardized direct associations in the first-order parallel model.

Path	*β*\beta*β*	SE	*p*
PDC → Motor control F1	0.652	0.068	<0.001
PDC → Negative experience processing F2	0.196	0.059	<0.001
PDC → Bodily self-representation F3	0.27	0.061	<0.001
PDC → Cognitive flexibility F4	0.33	0.05	<0.001
PDC → Mind–body integration F5	0.648	0.095	<0.001
Motor control F1 → PR	0.083	0.043	0.055
Negative experience processing F2 → PR	0.054	0.033	0.102
Bodily self-representation F3 → PR	0.301	0.045	<0.001
Cognitive flexibility F4 → PR	0.356	0.069	<0.001
Mind–body integration F5 → PR	0.037	0.024	0.115
PDC → PR	−0.012	0.045	0.794

PDC, perceived dance competence; PR, psychological resilience. Standard errors for some paths should be retained from the AMOS output when preparing the final table.

### 3.5 Indirect associations through embodied cognition dimensions

Indirect associations were tested using a bias-corrected bootstrap procedure with 5,000 resamples. The total indirect association between perceived dance competence and psychological resilience through the five embodied cognition dimensions was statistically significant, *β* = 0.287, SE = 0.050, 95% CI [0.20, 0.39], *p* < 0.001. Because the direct association between perceived dance competence and psychological resilience was non-significant, *β* = −0.012, p = 0.794, the observed pattern was consistent with an indirect association model. However, given the cross-sectional design, this pattern should not be interpreted as evidence of causal mediation.

The decomposition of specific indirect associations showed that cognitive flexibility had the largest specific indirect association, *β* = 0.117, 95% CI [0.07, 0.18], accounting for 40.8% of the total indirect association. Bodily self-representation had the second-largest specific indirect association, *β* = 0.081, 95% CI [0.04, 0.13], accounting for 28.2% of the total indirect association. Motor control also showed a smaller significant indirect association, *β* = 0.054, 95% CI [0.01, 0.10], accounting for 18.8% of the total indirect association.

By contrast, the specific indirect association through negative experience processing was not statistically significant, *β* = 0.011, 95% CI [−0.01, 0.04]. The specific indirect association through mind–body integration was also not statistically significant, *β* = 0.024, 95% CI [−0.01, 0.06]. These findings suggest that the observed indirect association between perceived dance competence and psychological resilience was mainly carried by cognitive flexibility and bodily self-representation, with motor control playing a smaller role.

The direct and indirect estimates had opposite signs, with a weak negative direct association and a positive total indirect association. This pattern is compatible with an inconsistent mediation structure. However, because the direct association was not statistically significant and the study was cross-sectional, the interpretation of suppression or competing pathways should be considered tentative.

**Figure 2:**
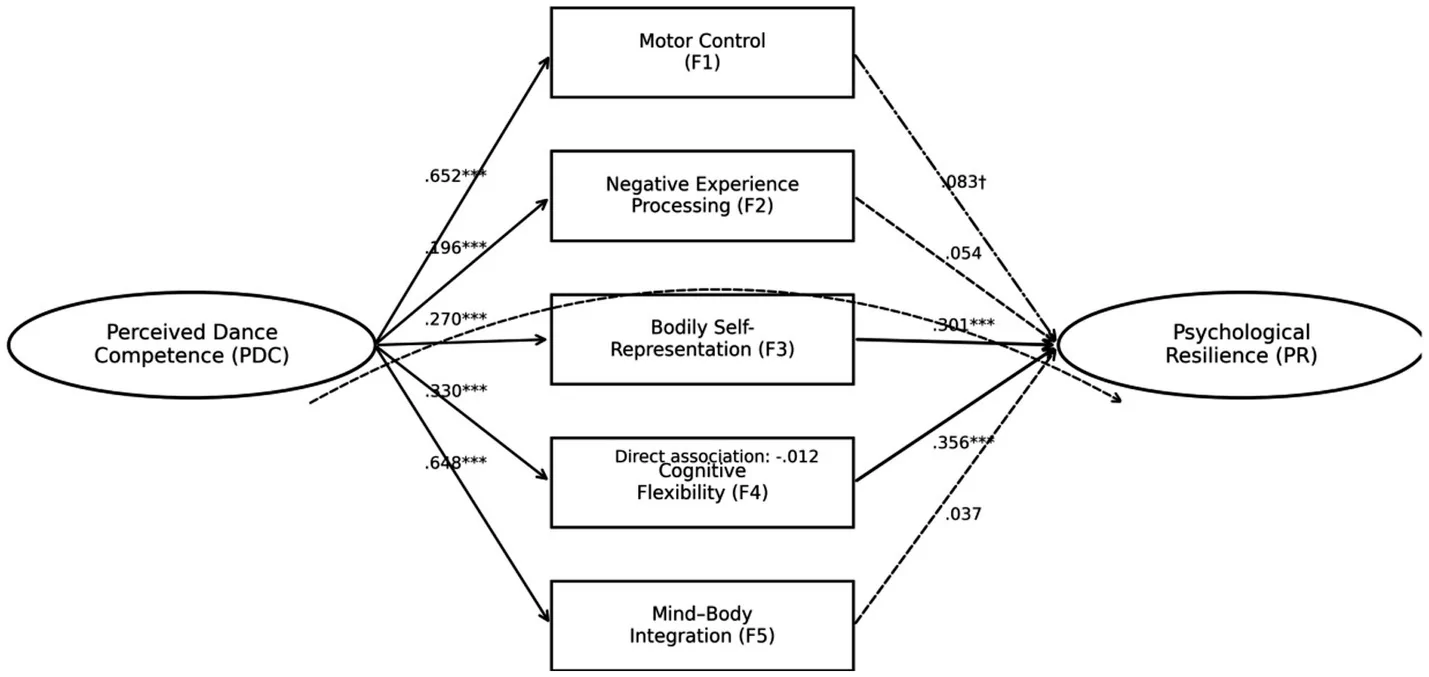
Standardized path estimates of the first-order parallel association model. PDC, perceived dance competence; PR, psychological resilience; F1, motor control; F2, negative experience processing; F3, bodily self-representation; F4, cognitive flexibility; F5, mind–body integration. Solid lines indicate statistically significant paths at (*p* < 0.001), the dash-dot line indicates a marginal path at (*p* < 0.10), and dashed lines indicate non-significant paths. The figure represents standardized associations rather than causal pathways.

The standardized path estimates of the first-order parallel association model are displayed in Figure 2. The figure illustrates the differentiated association pattern across the five embodied cognition dimensions: cognitive flexibility and bodily self-representation showed the strongest associations with psychological resilience, whereas negative experience processing and mind–body integration were not significantly associated with psychological resilience in the baseline model (Table 5).

**Table 5:** Decomposition of indirect associations between perceived dance competence and psychological resilience through embodied cognition dimensions (*N* = 303).

Indirect pathway	Stage 1 coefficient PDC → EC	Stage 2 coefficient EC → PR	Indirect association β\betaβ	Bootstrap 95% CI	Effect share
F1 Motor control	0.652^***^	0.083^†^	0.054	[0.01, 0.10]	18.80%
F2 Negative experience processing	0.196^***^	0.054	0.011	[−0.01, 0.04]	3.80%
F3 Bodily self-representation	0.270^***^	0.301^***^	0.081	[0.04, 0.13]	28.20%
F4 Cognitive flexibility	0.330^***^	0.356^***^	0.117	[0.07, 0.18]	40.80%
F5 Mind–body integration	0.648^***^	0.037	0.024	[−0.01, 0.06]	8.40%
Total indirect association	–	–	0.287	[0.20, 0.39]	100%
Direct association PDC → PR	–	–	−0.012	[−0.12, 0.09]	

PDC, perceived dance competence; EC, embodied cognition dimension; PR, psychological resilience. ^†^*p* < 0.10; ^***^p < 0.001. Because the study was cross-sectional, indirect associations should not be interpreted as evidence of causal mediation.

### 3.6 Explained variance

Perceived dance competence explained different proportions of variance across the five embodied cognition dimensions. The explained variance ranged from 13 to 42%, indicating that perceived dance competence was more strongly associated with some embodied cognition dimensions than others. The strongest associations were observed for motor control and mind–body integration, whereas weaker associations were observed for negative experience processing and bodily self-representation.

The five embodied cognition dimensions collectively explained 38% of the variance in psychological resilience. This result suggests that embodied cognition dimensions were meaningfully associated with resilience in the present sample. However, because the structural model showed suboptimal CFI and TLI values, the explained variance should be interpreted as preliminary evidence of association rather than as strong confirmation of the proposed theoretical model.

### 3.7 Robustness checks with covariates

To examine whether the core association pattern remained stable after accounting for demographic and training-related background variables, the structural model was re-estimated with gender, cumulative years of training, weekly training frequency, performance experience, and number of dance styles as covariates.

The covariate analysis showed that gender was significantly associated with perceived dance competence, *β* = −0.241, SE = 0.069, *p* < 0.001, indicating that male students reported higher perceived dance competence than female students under the coding scheme used in the dataset. Training duration, *β* = 0.098, *p* = 0.005, and performance experience, *β* = 0.156, *p* < 0.001, were also positively associated with perceived dance competence. Training frequency, *β* = 0.023, *p* = 0.336, and number of dance styles, *β* = 0.040, *p* = 0.066, were not significant predictors of perceived dance competence.

For psychological resilience, gender showed a significant independent association, *β* = 0.132, SE = 0.032, *p* < 0.001, indicating that female students reported higher psychological resilience than male students under the coding scheme used in the dataset. Training duration, *β* = −0.022, *p* = 0.150, training frequency, *β* = −0.005, *p* = 0.645, performance experience, *β* = −0.004, *p* = 0.736, and number of dance styles, *β* = −0.004, *p* = 0.635, were not significantly associated with psychological resilience after perceived dance competence and embodied cognition were included.

Importantly, the core structural pattern remained broadly stable after including covariates. Perceived dance competence remained significantly associated with motor control, bodily self-representation, and cognitive flexibility. Cognitive flexibility, bodily self-representation, and motor control remained the strongest dimensions associated with psychological resilience, whereas negative experience processing and mind–body integration remained non-significant. The direct association between perceived dance competence and psychological resilience also remained non-significant, *β* = 0.019, SE = 0.047, *p* = 0.677, 95% CI [−0.083, 0.123]. The total indirect association remained positive and statistically significant, *β* ≈ 0.30, with the 95% confidence interval excluding zero.

These robustness checks suggest that the observed indirect association pattern was not fully explained by demographic or training background variables. Nevertheless, the results remain limited by the cross-sectional self-report design, the preliminary psychometric status of the instruments, and the suboptimal incremental fit of the full structural model (Table 6).

**Table 6:** Effects of covariates on perceived dance competence and psychological resilience (*N* = 303).

Predictor	*β* (PDC)	SE	*p*	*β* (PR)	SE	*p*
Gender	−0.241	0.069	<0.001	0.132	0.032	<0.001
Training duration	0.098	0.035	0.005	−0.022	0.015	0.15
Training frequency	0.023	0.024	0.336	−0.005	0.01	0.645
Performance experience	0.156	0.03	<0.001	−0.004	0.013	0.736
Number of dance styles	0.04	0.022	0.066	−0.004	0.009	0.635

PDC, perceived dance competence; PR, psychological resilience.

### 3.8 Measurement invariance across dance disciplines

As an additional robustness check, measurement invariance was examined across the four major dance disciplines represented in the sample: Chinese classical dance, Chinese folk dance, ballet, and contemporary dance. The results supported configural and metric invariance, with ΔCFI values below 0.01. This suggests that the basic measurement structure and factor loadings were broadly comparable across dance disciplines.

However, given the modest sample size within some discipline subgroups, these invariance results should be treated as preliminary. Future studies with larger and more balanced samples across dance styles are needed to further examine whether the proposed measurement and structural patterns are stable across different dance genres.

## 4 Discussion

The present cross-sectional study examined the pattern of associations among perceived dance competence, multidimensional embodied cognition, and psychological resilience in undergraduate dance students. Specifically, the study investigated whether perceived dance competence was associated with multiple dimensions of embodied cognition, whether these embodied cognition dimensions showed differential associations with psychological resilience, and whether the association between perceived dance competence and psychological resilience was statistically accounted for by specific embodied cognition dimensions.

Overall, the findings provide preliminary support for a differentiated association pattern. Perceived dance competence was positively associated with all five embodied cognition dimensions, although the magnitude of these associations differed across dimensions. Cognitive flexibility and bodily self-representation showed the strongest associations with psychological resilience, whereas motor control showed a weaker and less stable association, and negative experience processing and mind–body integration were not significant predictors of resilience in the baseline model. The total indirect association from perceived dance competence to psychological resilience through embodied cognition dimensions was significant, whereas the direct association was weak and non-significant.

These findings should be interpreted cautiously. The study used cross-sectional self-report data, newly developed or contextually adapted instruments, and a structural model with suboptimal incremental fit indices. Therefore, the results should not be taken as evidence that perceived dance competence causes psychological resilience or that embodied cognition serves as a confirmed causal mechanism. Rather, the findings identify an associational pattern that is theoretically consistent with the possibility that body-mediated cognitive processes are relevant to resilience in dance contexts.

### 4.1 Directionality and causal interpretation

A central issue in interpreting the present findings concerns directionality. Although the model was specified according to the theoretical assumption that perceived dance competence is associated with psychological resilience through embodied cognition dimensions, the cross-sectional design cannot establish temporal order. Alternative explanations are therefore plausible.

For example, students with higher psychological resilience may be more likely to persist in demanding dance training, maintain confidence after setbacks, and evaluate their dance competence more positively. Dance training environments involve documented psychological pressures, including body image concerns, injury risk, evaluative anxiety, and emotional exhaustion (Walker and Nordin-Bates, 2010; Rice et al., 2016; Robazza et al., 2024; Smith et al., 2024). Students with greater pre-existing resilience may be better equipped to tolerate these stressors and remain engaged in training, thereby reporting stronger perceived competence and more adaptive embodied cognition. This possibility is consistent with sport and performance psychology research suggesting that resilience may shape how individuals respond to stressors and sustain participation in high-demand contexts (Fletcher and Sarkar, 2012; Sarkar and Fletcher, 2014; Gustafsson et al., 2011).

For this reason, the present findings should be understood as cross-sectional association patterns rather than causal evidence. Terms such as “indirect association” and “statistical mediation pattern” are used to describe the structure of observed covariation, not to claim temporal or causal mediation. Longitudinal, cross-lagged, experimental, and intervention-based designs are required to determine whether changes in perceived dance competence precede changes in embodied cognition and psychological resilience, or whether these variables influence one another reciprocally (MacKinnon et al., 2007; Hayes, 2018).

### 4.2 Dimensional specificity of embodied cognition

One important finding is that embodied cognition dimensions were not equally associated with psychological resilience. Cognitive flexibility and bodily self-representation showed the strongest positive associations with resilience. This pattern suggests that resilience in dance contexts may be more closely related to adaptive cognitive and self-representational processes than to movement control alone.

Cognitive flexibility may be relevant because dance students frequently need to respond to feedback, adjust movement strategies, reinterpret errors, and adapt to changing performance demands. In dance practice, movement difficulties, choreographic changes, bodily discomfort, and performance mistakes are common training experiences. Students who report greater cognitive flexibility may be more likely to interpret these experiences as manageable challenges rather than fixed failures. This interpretation is consistent with resilience perspectives that emphasize flexible adaptation, self-regulation, and adaptive appraisal under stress (Fletcher and Sarkar, 2012; Meichenbaum, 2007). It also aligns with embodied cognition perspectives suggesting that action, perception, and cognition are dynamically coordinated in adaptive behavior (Varela et al., 1991; Wilson, 2002; Shapiro, 2019). However, the present study cannot determine whether cognitive flexibility enhances resilience or whether resilient students tend to report greater cognitive flexibility.

Bodily self-representation was also strongly associated with psychological resilience. In dance, the body is not only an instrument of movement but also a central medium of self-expression, esthetic evaluation, and identity construction. A more coherent and positive representation of the body may be associated with greater confidence, self-continuity, and psychological stability in demanding training environments. This interpretation is consistent with dance psychology research emphasizing the importance of body image, bodily self-awareness, and self-perception in dancers’ psychological functioning (Quested and Duda, 2011; Walker and Nordin-Bates, 2010; Robazza et al., 2024). Nevertheless, the evidence remains correlational and self-reported.

Motor control showed a more limited association with psychological resilience. Perceived dance competence was strongly associated with motor control, but motor control was only marginally associated with resilience in the baseline model. This suggests that movement control may be closely tied to perceived dance competence but may not be the dimension most strongly related to resilience once other embodied cognition dimensions are considered. Rather than concluding that motor control is a necessary foundation for resilience, a more cautious interpretation is that motor control may be primarily competence-related, whereas its association with psychological adaptation may depend on broader cognitive and self-representational processes.

Negative experience processing and mind–body integration were not significant predictors of psychological resilience in the baseline model. These non-significant findings should not be overinterpreted. They may indicate that these dimensions were not directly associated with resilience in the present sample, but they may also reflect measurement limitations, restricted variance, or context-specific effects not captured in the current design. Previous work on body awareness and interoception suggests that bodily attention can be adaptive or maladaptive depending on how bodily signals are interpreted and regulated (Mehling et al., 2009; Mehling et al., 2012; Price and Hooven, 2018). Future studies should therefore examine whether negative bodily experience and mind–body integration operate through conditional, nonlinear, or context-dependent pathways.

### 4.3 Indirect association pattern

The total indirect association between perceived dance competence and psychological resilience through embodied cognition dimensions was significant. Among the five dimensions, cognitive flexibility accounted for the largest share of the total indirect association, followed by bodily self-representation and motor control. This pattern suggests that students who perceived themselves as more competent in dance also tended to report higher levels of certain embodied cognition dimensions, which in turn were associated with higher psychological resilience.

However, this result should not be interpreted as proof of causal mediation. In a cross-sectional design, indirect effects are statistical decompositions of covariance rather than evidence of temporal ordering or causal transmission (MacKinnon et al., 2007; Hayes, 2018). The present results indicate that the data are consistent with the proposed indirect association model, but they do not rule out reverse causality, reciprocal effects, omitted third variables, or shared method variance.

The weak and non-significant direct association between perceived dance competence and psychological resilience also requires cautious interpretation. Although the direct and indirect estimates had opposite signs, the direct path was not statistically significant. Therefore, it would be premature to conclude that perceived dance competence has a negative direct effect or that dance training involves a confirmed depletion pathway. The more appropriate interpretation is that perceived dance competence was not directly associated with psychological resilience after embodied cognition dimensions were included, whereas specific body-mediated cognitive dimensions were associated with resilience.

This finding supports a cautious theoretical proposition: perceived competence in dance may be psychologically relevant not simply because students feel technically capable, but because competence-related self-perceptions are associated with broader embodied cognition dimensions, particularly cognitive flexibility and bodily self-representation. Future longitudinal research is needed to test whether these dimensions mediate change over time.

### 4.4 Performing arts context and resilience

The findings also highlight the importance of studying resilience in performing arts contexts rather than assuming that models from competitive sport fully generalize to dance. Competitive sport often emphasizes external comparison, ranking, winning, losing, and outcome-based performance evaluation. Dance, by contrast, involves esthetic expression, bodily self-construction, emotional communication, and repeated refinement of movement quality (Quested and Duda, 2011; Walker and Nordin-Bates, 2010; Robazza et al., 2024).

This does not mean that dance is free from competition or external evaluation. Dance students may still experience auditions, examinations, performances, teacher evaluation, and peer comparison. However, the psychological demands of dance are often strongly tied to the body as an expressive and evaluative medium. Therefore, resilience in dance contexts may depend partly on how students perceive, regulate, and represent their bodies under pressure.

The present findings are consistent with this contextual interpretation. The strongest associations with psychological resilience were found for cognitive flexibility and bodily self-representation, rather than for perceived competence alone. This suggests that body-mediated self-perception and adaptive reinterpretation may be particularly relevant in esthetic and expressive movement settings. Nevertheless, this interpretation should be viewed as a theoretically informed reading of cross-sectional data rather than as a confirmed distinction between dance and sport populations. Direct comparative studies involving dancers, athletes, and non-dance students are needed to test whether the observed pattern is specific to dance.

### 4.5 Theoretical implications

The present study contributes to the literature in three cautious ways. First, it supports the value of treating embodied cognition as a multidimensional construct. The five embodied cognition dimensions did not show identical associations with psychological resilience. Cognitive flexibility and bodily self-representation were more strongly associated with resilience than negative experience processing or mind–body integration. This finding suggests that future research should avoid treating embodied cognition as a single undifferentiated variable, especially in movement-based contexts (Wilson, 2002; Shapiro, 2019; Mehling et al., 2012).

Second, the study provides preliminary evidence linking embodied cognition perspectives with resilience theory. Resilience theories often emphasize adaptive functioning, self-regulation, and flexible coping under stress (Fletcher and Sarkar, 2012; Sarkar and Fletcher, 2014). The present findings suggest that body-mediated cognitive processes may be relevant to these adaptive capacities among dance students. In particular, cognitive flexibility and bodily self-representation may represent promising constructs for future research on the embodied dimensions of psychological adaptation.

Third, the study extends discussion of resilience to the performing arts. Much resilience research in movement contexts has been shaped by competitive sport frameworks. By focusing on undergraduate dance students, the present study suggests that esthetic, expressive, and body-centered training contexts may involve distinctive psychological correlates. This does not replace existing sport psychology models but broadens the contexts in which resilience can be examined.

These contributions should be understood as preliminary. Because the measures require further validation and the structural model showed suboptimal incremental fit, the theoretical implications should be framed as directions for future investigation rather than definitive evidence for a new mechanism.

### 4.6 Practical implications

The practical implications of this study should be interpreted as hypotheses for future educational and intervention research rather than as direct recommendations. The findings suggest that cognitive flexibility and bodily self-representation may be promising targets for future dance-based psychological support programs. If replicated in longitudinal or experimental studies, dance education programs might consider incorporating reflective and adaptive movement activities that encourage students to reinterpret movement difficulties, respond flexibly to feedback, and develop a more coherent relationship with their bodies.

For example, future intervention studies could examine whether improvisational movement tasks, reflective body-awareness exercises, or guided performance reflection are associated with changes in cognitive flexibility, bodily self-representation, and psychological resilience. These possibilities are consistent with embodied and movement-based approaches that emphasize the role of bodily action, self-perception, and movement experience in psychological functioning (Koch et al., 2019; Price and Hooven, 2018; Shapiro, 2019). However, the present study did not test any intervention. Therefore, it cannot determine what type, duration, frequency, or intensity of dance-based activity would be effective.

The results may also be useful for educators in a general sense. Dance teachers and training programs may benefit from recognizing that students’ psychological adaptation is not necessarily linked only to technical performance. Students’ ability to flexibly interpret challenges and maintain a stable bodily self-concept may also be important. Even so, these implications should remain tentative until supported by longitudinal or experimental evidence.

### 4.7 Limitations and future research

Several limitations should be acknowledged. First, the study used a cross-sectional design. As a result, temporal order and causal direction cannot be established. The proposed model was theoretically specified, but the data cannot determine whether perceived dance competence precedes embodied cognition and resilience, whether resilience influences perceived competence, or whether these variables influence one another reciprocally. Future studies should use longitudinal panel designs, cross-lagged models, or experimental interventions to test temporal assumptions.

Second, all variables were measured using self-report questionnaires. This creates risks of common method variance, social desirability bias, and shared response tendencies. Although Harman’s single-factor test and the single-factor CFA suggested that common method variance was unlikely to fully explain the findings, these tests are limited. Future research should combine self-report measures with behavioral tasks, teacher ratings, physiological indicators, movement analysis, or performance-based assessments.

Third, the instruments used in the study were newly developed or contextually adapted. The D-CAPS, ECAS, and USTRS showed initial reliability and structural evidence, but they have not yet undergone sufficient independent validation. This is especially important because the theoretical model depends heavily on these measures. Future studies should test convergent validity, criterion validity, test–retest reliability, measurement invariance, and discriminant validity against established instruments. Additional evidence based on CR, AVE, and HTMT is useful but should not be treated as a substitute for independent validation and criterion-related evidence.

Fourth, the structural model showed suboptimal incremental fit indices. Although RMSEA and *χ*^2^/df suggested reasonable approximation, CFI and TLI were below conventional standards. Therefore, the SEM results should be interpreted as preliminary and exploratory rather than as strong confirmatory evidence. Future research should refine the measurement model, reduce construct overlap, test alternative specifications, and cross-validate the model in independent samples.

Fifth, the sample consisted of undergraduate dance students from one Chinese university. The findings may not generalize to professional dancers, recreational dancers, non-dance athletes, students from other cultural contexts, or individuals without systematic dance training. Future research should include larger, more diverse, and cross-cultural samples.

Sixth, the study did not directly compare dance students with athletes or non-dance students. Therefore, claims about the uniqueness of dance or performing arts contexts remain theoretical. Comparative studies involving competitive athletes, recreational exercisers, and non-dance performing artists would help clarify whether the observed association pattern is specific to dance.

Finally, the study did not test intervention effects. Therefore, educational implications should be treated as hypotheses. Future research should examine whether specific dance-based activities can produce measurable changes in embodied cognition and resilience over time.

## 5 Conclusion

This study provides preliminary cross-sectional evidence that perceived dance competence is associated with psychological resilience through specific dimensions of embodied cognition among undergraduate dance students. Cognitive flexibility and bodily self-representation showed the strongest associations with resilience, suggesting that adaptive cognitive regulation and body-related self-representation may be particularly relevant to psychological adaptation in dance contexts.

At the same time, the findings should be interpreted cautiously because of the cross-sectional design, self-report methodology, newly developed or adapted measures, and suboptimal structural model fit. The study does not establish that perceived dance competence causes resilience or that embodied cognition is a confirmed causal mechanism. Rather, it identifies an associational pattern that can guide future longitudinal, experimental, and measurement-validation research on the embodied dimensions of psychological resilience in performing arts education.

## Data Availability

The datasets presented in this study can be found in online repositories. The names of the repository/repositories and accession number(s) can be found at: Open Science Framework (OSF) – https://osf.io/mgbd8/overview?view_only=b1664f13fee24a78841d7af7664fb423.

## References

[ref1] Barsalou L. W. (2008). Grounded cognition. Annu. Rev. Psychol. 59, 617–645. doi: 10.1146/annurev.psych.59.103006.093639, 17705682

[ref2] Batson G. Sentler S. (2017). How visual and kinaesthetic imagery shape movement improvisation: a pilot study. J. Dance Somatic Pract. 9, 195–212. doi: 10.1386/jdsp.9.2.195_1

[ref3] Bläsing B. Calvo-Merino B. Cross E. S. Jola C. Honisch J. Stevens C. J. (2012). Neurocognitive control in dance perception and performance. Acta Psychol. 139, 300–308. doi: 10.1016/j.actpsy.2011.12.005, 22305351

[ref4] Brislin R. W. (1970). Back-translation for cross-cultural research. J. Cross-Cult. Psychol. 1, 185–216. doi: 10.1177/135910457000100301

[ref5] Canaipa R. Mendonça D. Agostinho M. Nascimento V. Honigman L. Treister R. (2021). En pointe: dancers report their pain less variably than do controls. J. Pain 22, 97–105. doi: 10.1016/j.jpain.2020.06.005, 32702405

[ref6] Christensen J. F. Cela-Conde C. J. Gomila A. (2017). Not all about sex: neural and biobehavioral functions of human dance. Ann. N. Y. Acad. Sci. 1400, 8–32. doi: 10.1111/nyas.13420, 28787539

[ref7] Connor K. M. Davidson J. R. T. (2003). Development of a new resilience scale: the Connor–Davidson resilience scale (CD-RISC). Depress. Anxiety 18, 76–82. doi: 10.1002/da.10113, 12964174

[ref8] Coubard O. A. Duretz S. Lefebvre V. Lapalus P. Ferrufino L. (2011). Practice of contemporary dance improves cognitive flexibility in aging. Front. Aging Neurosci. 3:Article 13. doi: 10.3389/fnagi.2011.00013 PMC3176453 21960971

[ref9] Cross E. S. Ticini L. F. (2012). Neuroaesthetics and beyond: new horizons in applying the science of the brain to the art of dance. Phenomenol. Cogn. Sci. 11, 5–16. doi: 10.1007/s11097-010-9190-y

[ref10] Dwarika M. S. Haraldsen H. M. (2023). Mental health in dance: a scoping review. Front. Psychol. 14:1090645. doi: 10.3389/fpsyg.2023.1090645, 36968742 PMC10035338

[ref11] Faul F. Erdfelder E. Buchner A. Lang A.-G. (2009). Statistical power analyses using G*power 3.1: tests for correlation and regression analyses. Behav. Res. Methods 41, 1149–1160. doi: 10.3758/BRM.41.4.1149, 19897823

[ref12] Fletcher D. Sarkar M. (2012). A grounded theory of psychological resilience in Olympic champions. Psychol. Sport Exerc. 13, 669–678. doi: 10.1016/j.psychsport.2012.04.007

[ref13] Fontanesi C. DeSouza J. F. X. (2021). Beauty that moves: dance for Parkinson’s effects on affect, self-efficacy, gait symmetry, and dual task performance. Front. Psychol. 11:Article 600440. doi: 10.3389/fpsyg.2020.600440, 33613357 PMC7892443

[ref14] Fornell C. Larcker D. F. (1981). Evaluating structural equation models with unobservable variables and measurement error. J. Mark. Res. 18, 39–50. doi: 10.2307/3151312

[ref15] Gustafsson H. Kenttä G. Hassmén P. (2011). Athlete burnout: an integrated model and future research directions. Int. Rev. Sport Exerc. Psychol. 4, 3–24. doi: 10.1080/1750984X.2010.541927

[ref16] Hayes A. F. (2018). Introduction to Mediation, Moderation, and Conditional process Analysis: A Regression-based Approach. 2nd Edn. New York, NY: Guilford Press.

[ref17] Henseler J. Ringle C. M. Sarstedt M. (2015). A new criterion for assessing discriminant validity in variance-based structural equation modeling. J. Acad. Mark. Sci. 43, 115–135. doi: 10.1007/s11747-014-0403-8

[ref18] Hu L.-T. Bentler P. M. (1999). Cutoff criteria for fit indexes in covariance structure analysis: conventional criteria versus new alternatives. Struct. Equ. Model. Multidiscip. J. 6, 1–55. doi: 10.1080/10705519909540118, 37339054

[ref19] Klaperski-van der Wal S. Skinner J. Opacka-Juffry J. Pfeffer K. (2025). Dance and stress regulation: a multidisciplinary narrative review. Psychol. Sport Exerc. 78:102823. doi: 10.1016/j.psychsport.2025.102823, 39922294

[ref20] Koch S. C. Riege R. F. F. Tisborn K. Biondo J. Martin L. Beelmann A. (2019). Effects of dance movement therapy and dance on health-related psychological outcomes: a meta-analysis update. Front. Psychol. 10:Article 1806. doi: 10.3389/fpsyg.2019.01806, 31481910 PMC6710484

[ref21] MacKinnon D. P. Fairchild A. J. Fritz M. S. (2007). Mediation analysis. Annu. Rev. Psychol. 58, 593–614. doi: 10.1146/annurev.psych.58.110405.085542, 16968208 PMC2819368

[ref22] Mehling W. E. Gopisetty V. Daubenmier J. Price C. J. Hecht F. M. Stewart A. (2009). Body awareness: construct and self-report measures. PLoS One 4:Article e5614. doi: 10.1371/journal.pone.0005614, 19440300 PMC2680990

[ref23] Mehling W. E. Price C. Daubenmier J. J. Acree M. Bartmess E. Stewart A. (2012). The multidimensional assessment of interoceptive awareness (MAIA). PLoS One 7:e48230. doi: 10.1371/journal.pone.0048230, 23133619 PMC3486814

[ref24] Meichenbaum D. (2007). “Stress inoculation training: a preventative and treatment approach,” in Principles and Practice of Stress Management, eds. Lehrer P. M. Woolfolk R. L. Sime W. E.. 3rd ed (New York, NY: Guilford Press), 497–518.

[ref25] Niedenthal P. M. (2007). Embodying emotion. Science 316, 1002–1005. doi: 10.1126/science.1136930, 17510358

[ref26] Pesce C. Tomporowski P. D. McCullick B. Pendleton D. M. Pesce C. (2015). Exercise and children’s cognition: the role of exercise characteristics and a place for metacognition. J. Sport Health Sci. 4, 47–55. doi: 10.1016/j.jshs.2014.09.003, 38826717

[ref27] Podsakoff P. M. MacKenzie S. B. Lee J.-Y. Podsakoff N. P. (2003). Common method biases in behavioral research: a critical review of the literature and recommended remedies. J. Appl. Psychol. 88, 879–903. doi: 10.1037/0021-9010.88.5.879, 14516251

[ref28] Price C. J. Hooven C. (2018). Interoceptive awareness skills for emotion regulation: theory and approach of mindful awareness in body-oriented therapy. Front. Psychol. 9:Article 798. doi: 10.3389/fpsyg.2018.00798 29892247 PMC5985305

[ref29] Quested E. Duda J. L. (2011). Antecedents of burnout among elite dancers: a longitudinal test of basic needs theory. Psychol. Sport Exerc. 12, 159–167. doi: 10.1016/j.psychsport.2010.09.003

[ref30] Rice S. M. Purcell R. De Silva S. Mawren D. McGorry P. D. Parker A. G. (2016). The mental health of elite athletes: a narrative systematic review. Sports Med. 46, 1333–1353. doi: 10.1007/s40279-016-0492-2, 26896951 PMC4996886

[ref9001] Robazza C. Vitali F. Bortoli L. Ruiz M. C. (2024). The role of mindfulness and motivational climate in athletes’ emotions: A person-centered approach. Front. Sports Active Living 6:1368399. doi: 10.3389/fspor.2024.1368399, 39744462 PMC11688466

[ref31] Sarkar M. Fletcher D. (2014). Psychological resilience in sport performers: a review of stressors and protective factors. J. Sports Sci. 32, 1419–1434. doi: 10.1080/02640414.2014.901551, 24716648

[ref32] Shapiro L. (2019). Embodied Cognition. 2nd Edn. New York, NY: Routledge.

[ref33] Smith L. S. Louw Q. A. Brink Y. (2024). Fatigue and recovery in ballet: exploring the experiences of professional south African ballet dancers. BMC Sports Sci. Med. Rehabil. 16:237. doi: 10.1186/s13102-024-01026-w, 39627829 PMC11616340

[ref34] Tomporowski P. D. Pesce C. (2019). Exercise, sports, and performance arts benefit cognition via a common process. Psychol. Bull. 145, 929–951. doi: 10.1037/bul0000200, 31192623

[ref35] Varela F. J. Thompson E. Rosch E. (1991). The Embodied mind: Cognitive Science and human Experience. Cambridge, Massachusetts: MIT Press.

[ref36] Walker I. J. Nordin-Bates S. M. (2010). Performance anxiety experiences of professional ballet dancers: the importance of control. J. Dance Med. Sci. 14, 133–145. doi: 10.1177/1089313X1001400402, 21703084

[ref37] Wilson M. (2002). Six views of embodied cognition. Psychon. Bull. Rev. 9, 625–636. doi: 10.3758/BF03196322, 12613670

[ref38] World Medical Association (2024). WMA Declaration of Helsinki: Ethical Principles for Medical Research Involving Human Participants. Ferney-Voltaire, France: World Medical Association. Available online at: https://www.wma.net/policies-post/wma-declaration-of-helsinki/ (Accessed October 15, 2025).

